# hsa_circ_0004276 inhibits osteogenic differentiation of bone marrow mesenchymal stem cells and exacerbates postmenopausal osteoporosis through interaction with ELAVL1

**DOI:** 10.1016/j.clinsp.2025.100718

**Published:** 2025-08-12

**Authors:** BaiQuan Fu, YuXin Sui, Yu Liu, XiaoTian Yang, Hui Leng

**Affiliations:** Department of Orthopaedics, Chifeng Municipal Hospital, Chifeng City, Inner Mongolia Autonomous Region, China

**Keywords:** hsa_circ_0004276, Postmenopausal osteoporosis, ELAVL1, Osteogenic differentiation, RNA-binding protein

## Abstract

**Objective:**

The aim of this study was to investigate the potential of hsa_circ_0004276 as a diagnostic marker for Postmenopausal Osteoporosis (PMO) and its potential interaction with the RNA-binding protein ELAV-like RNA binding protein-1 (ELAVL1).

**Methods:**

The circRNA expression profiles obtained from the GSE159121 dataset were used to screen for differentially expressed circRNAs in patients with PMO. hsa_circ_0004276 was further analyzed and validated for its circular structure using a bioinformatics website, actinomycin D, and RNase R assays. Serum samples from 115 PMO patients and 103 healthy controls were collected. RT-qPCR and western blot were applied to assess hsa_circ_0004276 and ELAVL1. The effects of hsa_circ_0004276 and ELAVL1 on osteogenic differentiation of human Bone Marrow Mesenchymal Stem Cells (BMSCs) were investigated by in vitro experiments.

**Results:**

hsa_circ_0004276 was highly expressed in PMO patients and negatively correlated with low bone mineral density. hsa_circ_0004276 had high specificity and sensitivity for the diagnosis of PMO. hsa_circ_0004276 significantly interacted with ELAVL1. During BMSC osteogenic differentiation, hsa_circ_0004276 knockdown significantly increased calcium deposition and ALP activity, while hsa_circ_0004276 overexpression showed the opposite effect. In addition, ELAVL1 expression was reduced in PMO patients and positively correlated with hsa_circ_0004276.

**Conclusion:**

hsa_circ_0004276 regulates osteogenic differentiation of BMSCs through its interaction with ELAVL1 in PMO pathogenesis. This finding provides a new perspective for the study of the molecular mechanism of PMO and a theoretical basis for the development of new therapeutic targets.

## Highlights

• Identification of aberrantly expressed circRNAs in PMO.
• Increased expression of hsa_circ_0004276 during OD of BMSCs.
• ELAVL1 inhibits the OD of BMSCs.

## Introduction

Postmenopausal Osteoporosis (PMO) is a systemic bone disease that primarily affects postmenopausal women.,^1^ It is caused by increased bone resorption, decreased bone formation, and imbalances in calcium metabolism due to significantly lower estrogen levels. It is also exacerbated by genetic factors, poor lifestyles such as low-calcium diets, physical inactivity, smoking, and excessive alcohol consumption.^2^, ^3^, ^4^ The diagnosis of PMO is primarily made by evaluating Bone Mineral Density (BMD) by dual-energy X-Ray absorptiometry.^5^^,^^6^ Treatment strategies include medication, increased calcium and vitamin D intake, moderate physical activity, pain management, and fracture prevention.^7^, ^8^, ^9^

Osteoporosis is closely associated with dysfunction of Bone Marrow Stromal Cells (BMSCs) that participate in Osteogenic Differentiation (OD).^10^ BMSCs can differentiate into osteoblasts, a process that is regulated by a variety of cytokines, hormones, and gene regulatory networks.^11^ In the osteoporotic state, the OD ability of BMSCs is impaired due to factors such as changes in hormone levels, increased inflammatory factors, enhanced oxidative stress, and nutritional deficiencies, leading to a decrease in the number and function of osteoblasts.^12^^,^^13^ In addition, these factors may also stimulate the differentiation of BMSCs towards adipocytes, further reducing osteoblast formation.^14^ Thus, impaired OD of BMSCs not only reduces the rate of bone formation but may also increase the risk and severity of osteoporosis. Therefore, improving the OD ability of BMSCs or modulating their differentiation direction may provide new strategies for the prevention and treatment of osteoporosis. circRNAs are a class of closed-loop structured non-coding RNAs with a unique morphology that does not contain free 5′ and 3′ ends, thus exhibiting a high degree of stability in cells.^15^ These RNAs are formed by splicing of precursor mRNAs and were initially thought to be the product of splicing errors, but are now known to play roles in gene expression regulation, miRNA sponging, and participation in protein coding.^16^ Several works have confirmed the involvement of circRNAs in the regulation of PMO as well as OD of BMSCs. For example, circRNA_0048211 protects against PMO by targeting miR-93–5p to regulate BMP2.^17^ Postmenopausal Chinese Han women with dysregulation of circ_0076906 and circ_0134944 have an increased risk for osteoporosis and osteoporotic fractures.^18^ hsa_circ_0006215 regulates RUNX2 through competitive binding to miR-942–5p and VEGF to promote OD of BMSCs and enhance osteogenic-vascular coupling.^19^ Considering that there are many members of the circRNA family, and the roles of different circRNAs in PMO and OD are quite different, identifying and determining circRNAs that regulate the OD of BMSCs is an important step towards understanding PMO pathogenesis.

In this work, the authors aimed to probe the circRNAs that are aberrantly expressed in PMO and to reveal circRNAs that can serve as potential biomarkers for PMO diagnosis. In addition, RNA-binding proteins that interact with hsa_circ_0004276 were probed by in vitro experiments.

## Materials and methods

### Bioinformatics analysis

The GSE159121 dataset provided circRNA expression profiles of peripheral blood from three PMO patients and healthy patients on the annotation platform GPL16791. These expression profiles were analyzed using by R language software. Genes in the GSE159121 database were analyzed, with those with Log2 Fold change > 1 and *p* < 0.05 were labeled as differential genes. The interaction of hsa_circ_0004276 with the RNA-binding protein ELAV-like RNA binding protein-1 (ELAVL1) was obtained from the bioinformatics website circbase (https://rnasysu.com/encori/index.php). circPrism software and circbank website (http://www.circbank.cn/index.html) were used to analyze the genetic information of hsa_circ_0004276.

### Clinical samples

The study was approved by the Ethics Committee of Chifeng Municipal Hospital. All patients participating in the study provided informed consent. This study follows the STROBE guidelines. Serum samples from 115 PMO patients were obtained from Chifeng Municipal Hospital during 2021‒2023. The patients were diagnosed with osteoporosis on the basis of BMD measured by dual-energy X-ray absorptiometry. Exclusion criteria were as follows: use of corticosteroids, history of fractures, menopause before 40-years of age, hyperthyroidism, rheumatoid arthritis, malignancy, renal failure or disease, oral bisphosphonates within the past 6-months, chronic liver disease, and hyperparathyroidism. In addition, serum samples from 108 healthy people were collected as controls. Table 1 shows demographic and anthropometric information on all patients.

**Table 1:** Clinical information table of subjects.

Characteristic	Control (*n* = 108)	PMO (*n* = 115)	p-value
Age (yeas)	56.23 ± 13.23	57.82 ± 9.84	0.528
BMI (kg/m^2^)	33.46 ± 4.21	27.64 ± 2.17	<0.0001
smoking	10	18	0.861
Non-smoking	23	34	
Vitamin D3 (ng/mL)	45.84 ± 17.94	43.27 ± 22.21	0.578
DII	−0.17 ± 1.13	0.74 ± 1.24	0.001
L.S BMD (g/cm^2^)	1.14 ± 0.17	0.69 ± 0.21	<0.0001
L.S T-score	0.07 ± 0.43	−2.74 ± 0.84	<0.0001
L.S Z-score	0.97 ± 1.13	−1.04 ± 0.56	<0.0001
F.N BMD (g/cm^2^)	0.91 ± 0.17	0.68 ± 0.13	<0.0001
F.N T-score	0.31 ± 0.74	−1.01 ± 0.47	<0.0001
F.N Z-score	1.24 ± 0.93	−0.83 ± 0.47	<0.0001

BMI, Body Mass Index; PMO, Postmenopausal Osteoporosis; DII, Dietary Inflammatory Index; L.S BMD, Lumbar Spine Bone Mineral Density; L.S T-score, Lumbar Spine T-score; L.S Z-score, Lumbar Spine Z-score; F.N BMD, Femoral Neck Bone Mineral Density; F.N T-score, Femoral Neck T-score; F.N Z-score, Femoral Neck Z-score.

### Cell culture

Human BMSCs (SALIAI, Guangzhou, China) were cultured in DMEM/F12 (GIBCO; A4192001) supplemented with 10 % fetal bovine serum (FBS; GIBCO, 10099141C), 100 U/mL penicillin, and 100 U/mL streptomycin and placed in a 37 °C, 5 % CO_2_ incubator. Cells were dissociated by trypsin when their density reached about 80 %, and then inoculated in 6-well plates for other experiments.

### hsa_circ_0004276 ring structure assays

BMSCs were inoculated in six-well plates (5 × 10^5^ cells/well). After 24 h, cells were exposed to 2 μg/mL actinomycin D (Sigma) and collected at the indicated time points. hsa_circ_0004276 and linear GAPDH mRNA stability were analyzed using RT-qPCR.

BMSCs were treated with RNase R (4 U/mg, Epicenter) and incubated at 37 °C for 30 min. Then, the treated RNA was reverse transcribed with specific primers, and hsa_circ_0004276 and linear GAPDH mRNA were detected by quantitative real-time PCR assay.

### Gel electrophoresis

To create a 1 % agarose gel, 50 mL of 1 × TAE buffer was mixed with 0.5 g of agarose and heated until boiling. Post cooling down to a temperature range of 70°‒80 °C, 5 μL of 4S GelRed (Sangon, Shanghai, China) was mixed. Following the stabilization of the perfusion solution, the gel was transferred into an electrophoresis container containing 1 × TAE buffer. Following this, each well contributed 10 μL of DNA samples, which were then combined with the sampling buffer. The electrophoresis process took place at 120 V for half an hour, with the resulting bands on the gel being recorded through a UV imaging technique.

### Cell transfection

The siRNA and pcDNA 3.1 overexpression vectors targeting hsa_circ_0004276 and ELAVL1 (GenePharma, Shanghai, China) were transiently transfected into BMSCs using Lipofectamine 3000 (Invitrogen). After 48 h of transfection, transfection efficiency was assessed by RT-qPCR or western blot.

### Osteogenic induction

BMSCs were cultured on adherent walls in 6-well plates. OD of BMSCs was induced using an osteoblast differentiation kit (#HuxMA-90,021, Cyagen, Guangzhou, China). BMSCs were collected at 0d, 7d, 14d, and 21d of induction. The culture medium was changed every 3d The kit consisted of Cell Culture Basal Medium (177 mL; BLDM-03,011), FBS (Superior-Quality) (20 mL; FBSSR-01,021), and Supplement for OD of human BMSCs (3 mL; huxmx-04,021).

### Alizarin red staining

BMSCs were cultured at 1 × 10^5^ cells/well in osteogenic induction medium for 2 weeks. After rinsing, BMSCs were fixed with 10 % paraformaldehyde for 10 min. A 1 % alizarin red solution (Sigma-Aldrich) was prepared and stained for 5 min. After rinsing again, calcium nodules were visualized with an inverted light microscope (Image-Pro Plus 6.0, Media Cybernetics, USA).

### ALP staining

ALP-positive cells were detected using an ALP staining kit (Thermo Fisher Scientific, Inc.).^20^ Briefly, BMSCs were fixed with 4 % paraformaldehyde and incubated with substrate solution at 37 °C for 1 hour to allow ALP reaction. ALP staining was detected using a digital camera and colorimetric detector (ProteinSimple Inc., USA).

### Adipogenic induction

BMSCs were seeded at a density of 2 × 10^4^ cells/cm^2^ in 6-well plates and cultured to 80 %‒90 % confluence. Adipogenic differentiation was induced using a human BMSC adipogenic differentiation kit (#HuxMA-90,031, Cyagen, Guangzhou, China). The differentiation protocol followed a cyclic induction/maintenance approach. Induction medium (Adipogenic Differentiation Medium A) was applied for 3 days, followed by 1 day in maintenance medium (Adipogenic Differentiation Medium B). This cycle was repeated for three rounds (12 days total), after which cells were cultured in maintenance medium until day 21. The culture medium was changed every 3 days.

### Oil red O staining

To evaluate lipid droplet formation, BMSCs were fixed with 4 % paraformaldehyde (Sigma-Aldrich) for 30 min at room temperature and stained with Oil Red O working solution (0.5 % w/v in isopropanol, diluted 3:2 with distilled water; Sigma-Aldrich, O0625) for 30 min. Excess stain was removed by washing with PBS, and cells were imaged using an inverted microscope (Olympus IX73, Tokyo, Japan). For quantification, Oil Red O was extracted with 100 % isopropanol, and absorbance was measured at 510 nm using a microplate reader (BioTek Synergy H1, Winooski, VT, USA).

### RT-qPCR

Total RNA was extracted from serum and BMSCs with TRIzol reagent (15596018, Thermo Fisher Scientific) and then reverse transcribed to cDNA with PrimeScript RT master mix (RR058A, Takara, Japan). SYBR Green qPCR Super Mix-UDG (Thermo Fisher) was applied to perform RT-qPCR. GAPDH was taken as an endogenous reference gene. Gene expression was calculated based on the 2^-ΔΔCT^ method.

### Western blot

Samples were lysed using RIPA lysis buffer (Beyotime) containing a mixture of protease inhibitors. Total protein was quantified by the BCA kit (Beyotime Biotechnology, China). Protein suspensions were loaded into SDS-PAGE, separated, and electrophoretically transferred to a polyvinylidene difluoride membrane using an electroblotting device (Bio-Rad, USA). Membranes were closed in 5 % bovine serum albumin for 1 h and then incubated with the primary antibody DMT1 (15083, Cell Signaling Technology). GAPDH (2118, Cell Signaling Technology) was taken as a reference control. After incubation with HRP-coupled secondary antibody for 1 h, protein signals on the membranes were detected using a gel imaging scanning system (Bio-Rad) and analyzed by ImageJ.

### RIP assay

The Magna RIP kit (Millipore, USA) was used here for RIP assays. BMSCs were lysed and then exposed to ELAVL1 antibody or IgG antibody-coupled magnetic beads. RNA complexes on the magnetic beads were eluted to isolate total RNA. RT-qPCR was performed to detect RNA expression.

### Data analysis

Data were expressed as mean ± Standard Deviation (SD). The biological replicates of all experiments were at least three. Statistical analysis was performed using GraphPad Prism 9.0 (GraphPad Software, USA). The sensitivity and specificity of circRNA in the diagnosis of PMO were determined by ROC. The Shapiro-Wilk test was employed to assess the normal distribution of the data. Count data were assessed using the Chi-Square test. Two-group comparisons were performed using Student's *t*-test, and multigroup comparisons were performed using one-way ANOVA and Tukey HSD. * *p* < 0.05 was considered statistically different.

## Results

### Identification of aberrantly expressed circRNAs in PMO

**Fig. 1:**
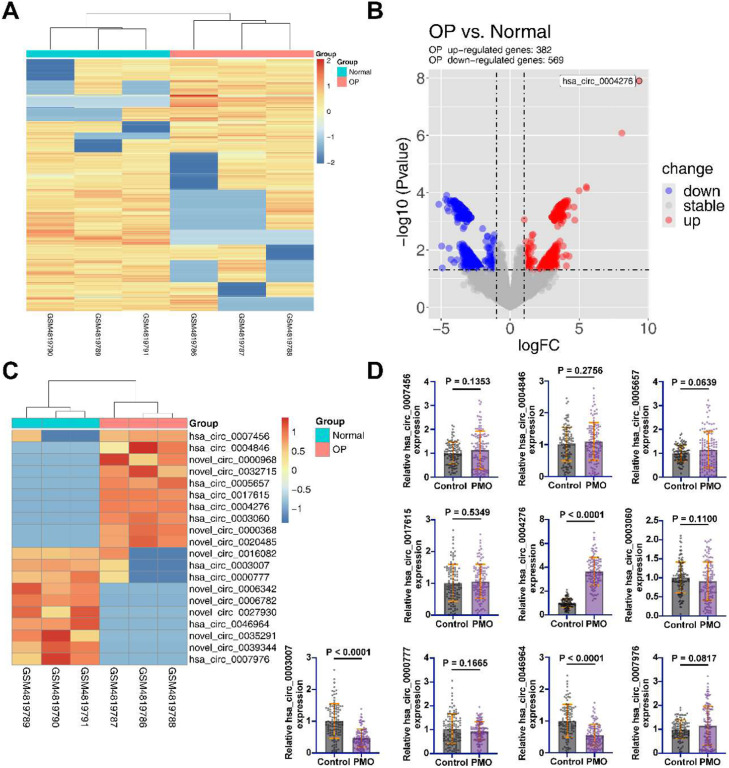
Identification of aberrantly expressed circRNAs in the PMO. (A) Heatmap showing the top 500 most abundant circRNAs in the dataset GSE159121; (B) Volcano plot showing the abnormally highly expressed and abnormally low expressed circRNAs in the dataset GSE158695; (C) Heatmap showing the top 10 most highly expressed and lowly expressed circRNAs in the dataset GSE158695; (D) RT-qPCR for the expression of aberrantly expressed circRNAs in human samples; data are expressed as mean ± SD (*p < 0.05).

To explore the aberrantly expressed circRNAs in PMO, the authors selected GSE datasets from the GEO database (GSE159121) and performed expression profiling of the differential circRNAs in these datasets by R language software. The heatmap demonstrated the top 500 most abundant circRNAs (Fig. 1A). A total of 569 abnormally low-expressed circRNAs and 382 abnormally high-expressed circRNAs were identified (Fig. 1B, Supplementary Table 1). Subsequently, the authors analyzed the top 10 highly expressed and low-expressed circRNAs, of which 10 circRNAs were newly identified circRNAs (Fig. 1C). They were circ_0000968, circ_0032715, circ_0000368, circ_0020485, circ_0016082, circ_0006342, circ_0006782, circ_0027930, circ_0035291, and circ_0039344. Considering that the sequence information of these 10 novel circRNAs was not found in open databases to design effective primers for PCR analysis, only the remaining 10 circRNAs were analyzed by PCR. Among the 10 identified circRNAs, the authors found that hsa_circ_0004276 was up-regulated in PMO patients, while hsa_circ_0003007 and hsa_circ_0046964 were abnormally down-regulated in PMO patients (Fig. 1D). And hsa_circ_0004276 was the highest differentially expressed circRNA. Therefore, hsa_circ_0004276 was selected for subsequent studies.

### Identification of hsa_circ_0004276 loop structure

**Fig. 2:**
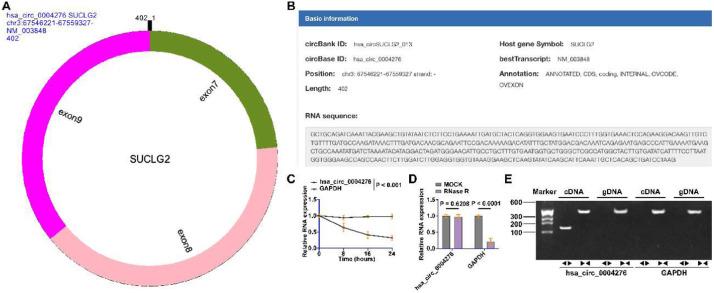
Identification of hsa_circ_0004276 ring structure. (A) CircPrism software predicted the ring structure of hsa_circ_0004276; (B) Bioinformatics website circbank queried the gene information of hsa_circ_0004276; (C) Actinomycin D assay detected the ring structure of hsa_circ_0004276; (D) RNase R assay detected hsa_ circ_0004276 resistance to RNase R digestion; (E) Gel electrophoresis to detect the ring structure of hsa_circ_0004276; data are expressed as mean ± SD (*p < 0.05).

Subsequently, the authors analyzed by circPrism software and the bioinformatics website circbank (http://www.circbank.cn/index.html) and found that hsa_circ_0004276 is composed of exons 7‒9 of SUCLG2, located in chr3: 67,546,221–67,559,327 strand:, with a length of 402 bp (Fig. 2A‒B). Subsequently, the authors evaluated the loop structure of hsa_circ_0004276. Actinomycin D did not affect the stability of hsa_circ_0004276, but significantly inhibited the stability of GAPDH mRNA (Fig. 2C). In addition, RNase R could not digest hsa_circ_0004276, but GAPDH mRNA (Fig. 2D). To ensure that hsa_circ_0004276 was spliced from end to end rather than trans-spliced or genomic rearrangement, divergent and convergent primers were designed. PCR results showed that hsa_circ_0004276 was only detected in cDNA, thus ruling out the presence of hsa_circ_000427 in gDNA, whereas the convergent primers amplified GAPDH in both cDNA and gDNA (Fig. 2E).

### hsa_circ_0004276 is associated with BMD in PMO patients

**Fig. 3:**
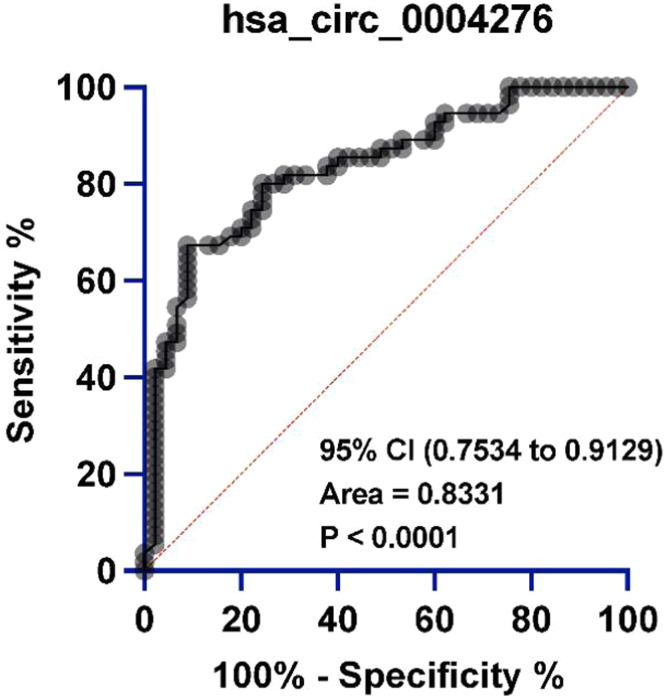
Specificity and sensitivity of hsa_circ_0004276 in the diagnosis of PMO analyzed by ROC curve.

BMD of femoral neck and lumbar spine in PMO patients was significantly lower than that of healthy patients (Table 1). After adjusting for age, BMI, and vitamin D3 levels, Lumbar Spine Bone Mineral Density (L.S BMD) remained significantly lower in the PMO group than in the control group (β = −0.41, 95 % CI [−0.51, −0.31] *p* < 0.001) (Table 2). In addition, the dietary inflammatory index was higher in PMO patients than in healthy patients. In addition, the ROC curve results indicated that serum hsa_circ_0004276 had high sensitivity and specificity for the clinical diagnosis of PMO (Fig. 3).

**Table 2:** Multiple linear regression analysis for predicting Lumbar Spine Bone Mineral Density (L.S BMD).

Characteristic	Regression coefficient (β)	Standard error (SE)	95 % CI	p-values
Groups (PMO vs. Control)	−0.41	0.051	−0.51, −0.31	<0.001
Age (years)	−0.005	0.002	−0.009, −0.001	0.015
BMI (kg/m^2^)	0.015	0.0026	0.010, 0.020	<0.001
Vitamin D3 (ng/mL)	0.001	0.0008	−0.0005, 0.0025	0.150

BMI, Body Mass Index; PMO, Postmenopausal Osteoporosis.

### Increased expression of hsa_circ_0004276 during OD of BMSCs

**Fig. 4:**
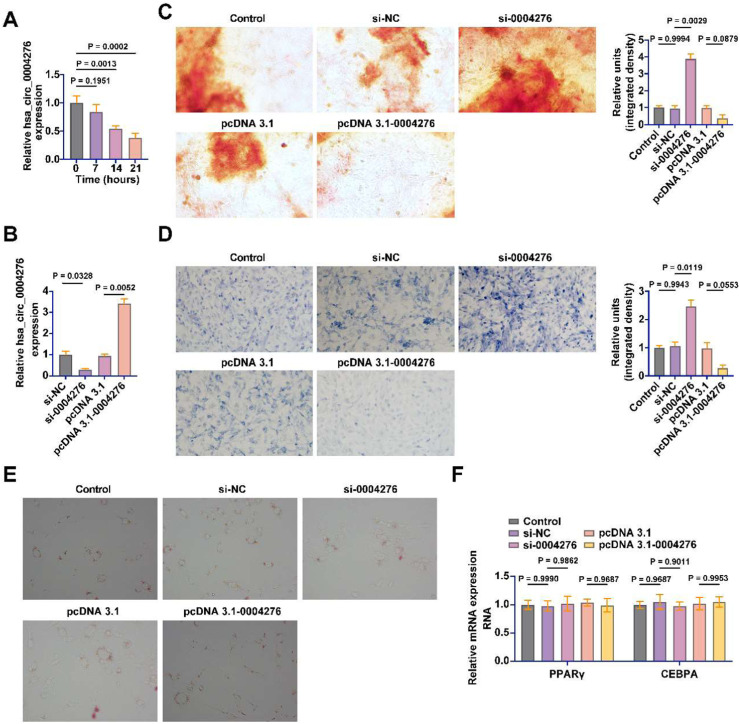
Increased expression of hsa_circ_0004276 during OD of BMSCs. (A) RT-qPCR to detect the expression changes of hsa_circ_0004276 during OD of BMSCs; (B) RT-qPCR to detect the transfection efficiency of si-hsa_circ_0004276 and pcDNA 3.1-hsa_circ_0004276; (C) Alizarin red staining to detect calcium deposition in OD of BSMCs; (D) ALP staining to detect ALP level in OD of BMSCs; (E) Oil red O staining to detect the induction of adipogenic differentiation of BMSCs; (F) RT-qPCR to detect the mRNA expression of PPARγ and CEBPA in adipogenic differentiation of BMSCs; data are expressed as mean ± SD (*n* = 3). *p < 0.05.

Subsequently, the authors explored the potential molecular mechanisms by which hsa_circ_0004276 is associated with BMD. hsa_circ_0004276 expression decreased in a time-dependent manner during OD of BMSCs (Fig. 4A). Subsequently, by transfecting siRNA and pcDNA 3.1 overexpression vector, the authors knocked down and overexpressed hsa_circ_0004276 in BMSCs, respectively (Fig. 4B). Alizarin red staining showed that hsa_circ_0004276 knockdown significantly increased calcium deposition during OD of BMSCs, but hsa_circ_0004276 overexpression showed the opposite effect (Fig. 4C). ALP staining showed that hsa_circ_0004276 knockdown effectively increased ALP activity during OD, but hsa_circ_0004276 overexpression inhibited ALP activity (Fig. 4D). The effect of hsa_circ_0004276 on adipogenic differentiation of BMSCs was further evaluated. Knockdown or overexpression of hsa_circ_0004276 did not affect the formation of lipid droplets during adipogenic differentiation of BMSCs, nor did it affect the mRNA expression of PPARγ and CEBPA (Fig. 4E‒F).

### hsa_circ_0004276 interacts with ELAVL1

**Fig. 5:**
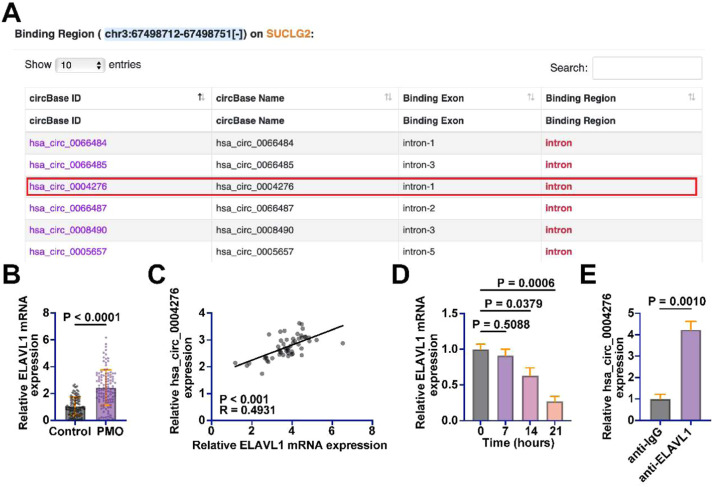
hsa_circ_0004276 interacts with ELAVL1. (A) Bioinformatics website starbase queried RBPs with potential interactions with hsa_circ_0004276; (B) RT-qPCR detected ELAVL1 expression in serum of PMO patients and healthy patients; (C) Pearson's correlation analysis to assess the correlation between ELAVL1 and hsa_circ_0004276 in PMO; (D) RT-qPCR to detect changes in ELAVL1 expression during OD of BMSCs; (E) RIP assay to detect the interaction between ELAVL1 and hsa_circ_0004276; data are expressed as mean ± SD (*n* = 3). *p < 0.05.

Many circRNAs regulate the availability and function of these proteins by binding to RBPs. When circRNAs bind RBPs, they can prevent these proteins from interacting with other target RNAs, thereby affecting the processing, stability, and translation of the latter. Subsequently, the authors explored RNA-binding proteins that potentially interact with hsa_circ_0004276. In the preliminary exploration, the authors queried the RBPs that potentially bind to hsa_circ_0004276 through the bioinformatics website Starbase (https://rnasysu.com/encori/index.php) and found that hsa_circ_0004276 has potential binding relationships with multiple RBPs (Fig. 5A). Among them, the authors focused on ELAVL1. ELAVL1 has been found to have an inhibitory effect on bone formation in diabetic osteoporotic mice.^21^ ELAVL1 expression in the serum of PMO patients was higher than that of healthy patients (Fig. 5B). ELAVL1 and hsa_circ_0004276 levels in the serum of PMO patients were positively correlated (Fig. 5C). In addition, ELAVL1 expression was abnormally reduced during the induction of OD in BMSCs (Fig. 5D). Subsequently, the interaction of ELAVL1 with hsa_circ_0004276 was detected by RIP assay, showing that anti-ELAVL1 significantly enriched hsa_circ_0004276 compared with anti-IgG (Fig. 5E).

### ELAVL1 inhibits OD of BMSCs

**Fig. 6:**
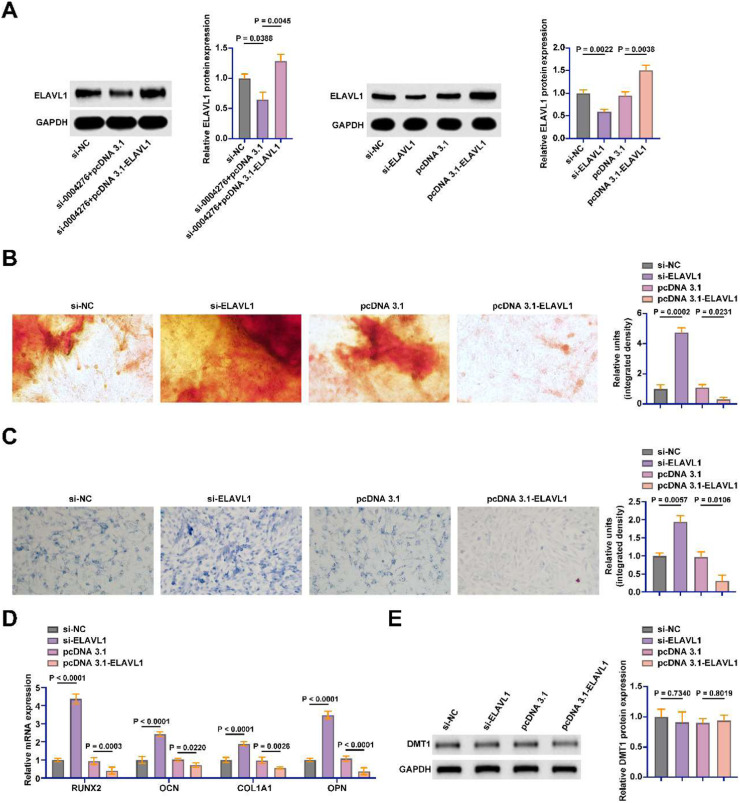
ELAVL1 inhibits OD of BMSCs. The siRNA and pcDNA 3.1 overexpression vector of ELAVL1 were transfected into BMSCs. (A) Western blot to detect the protein expression of ELAVL1; (B) Alizarin red staining to detect the changes of calcium deposition in OD of BSMCs; (C) ALP staining to detect the changes of ALP in OD of BMSCs; (D) RT-qPCR to detect the mRNA expression of RUNX2, OCN, COL1A1 and OPN in BMSCs; (E) Western blot to detect the expression of DMT1 in BMSCs; data are expressed as mean ± SD (*n* = 3). *p < 0.05.

Subsequently, the authors transfected the siRNA and pcDNA 3.1 overexpression vector of ELAVL1 into BMSCs. siRNA and pcDNA 3.1 overexpression vectors successfully knocked down and overexpressed ELAVL1 in BMSCs, respectively (Fig. 6A). ELAVL1 knockdown promoted calcium deposition and ALP activity during OD, while ELAVL1 overexpression inhibited calcium deposition and ALP activity (Fig. 6B‒C). The osteogenic marker genes were further detected by RT-qPCR. Knockdown of ELAVL1 significantly promoted the mRNA expression of RUNX2, OCN, COL1A1, and OPN in BMSCs, but overexpression of ELAVL1 had the opposite effect (Fig. 6D). DMT1 was found to be a downstream gene of ELAVL1 in diabetic osteoporosis. Knockdown or overexpression of ELAVL1 did not affect the protein expression of DMT1 in BMSCs (Fig. 6E).

### hsa_circ_0004276 inhibits BMSC OD by regulating ELAVL1

**Fig. 7:**
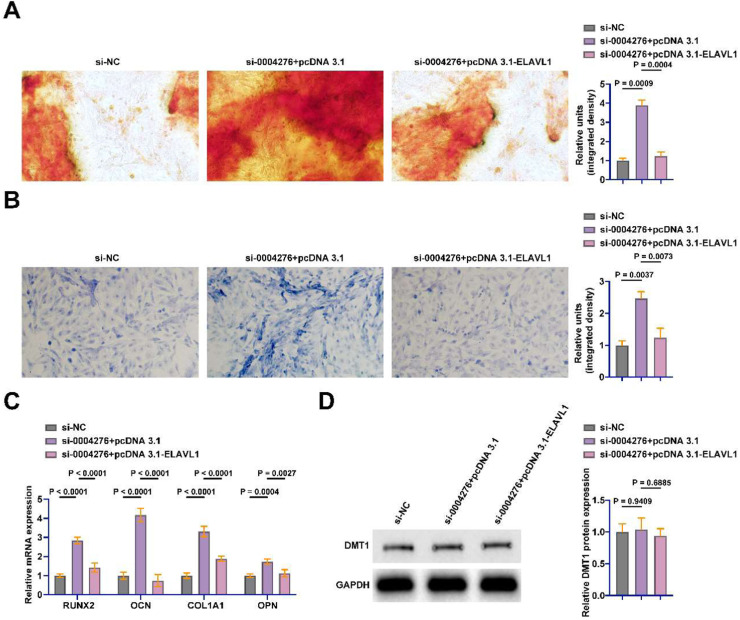
hsa_circ_0004276 inhibits BMSC OD by regulating ELAVL1. si-hsa_circ_0004276 and pcDNA 3.1-ELAVL1 were co-transfected into BMSCs. (A) Alizarin red staining to detect the changes of calcium deposition in OD of BSMCs; (B) ALP staining to detect the changes of ALP in OD of BMSCs; (C) RT-qPCR to detect the mRNA expression of RUNX2, OCN, COL1A1 and OPN in BMSCs; (D) Western blot to detect the expression of DMT1 in BMSCs; data are expressed as mean ± SD (*n* = 3). *p < 0.05.

The authors cotransfected si-hsa_circ_0004276 and pcDNA 3.1-ELAVL1 into BMSCs. hsa_circ_0004276 knockdown significantly promoted calcium deposition and ALP activity during OD, but ELAVL1 overexpression impaired this process (Fig. 7A‒B). In addition, knockdown of hsa_circ_0004276 significantly promoted the mRNA expression of RUNX2, OCN, COL1A1, and OPN in BMSCs, but this effect was reversed by overexpression of ELAVL1 (Fig. 7C). Western blot showed that knockdown of hsa_circ_0004276 and overexpression of ELAVL1 did not affect the protein expression of DMT1 in BMSCs (Fig. 7D).

## Discussion

circRNA has garnered increasing attention from researchers across a variety of diseases, including cancer,^22^ osteoporosis,^23^ diabetes,^24^ and others. However, the mechanisms by which different circRNAs contribute to PMO vary substantially, underscoring the critical need to further explore their roles in PMO to deepen the understanding of its pathogenesis and progression. circRNAs possess a stable circular structure, making them resistant to degradation and thus promising candidates as reliable biomarkers.^25^ While many studies have focused on the role of circRNAs in sequestering downstream miRNAs,^26^ the interaction between circRNAs and RBPs in PMO remains underexplored. In this study, the authors found that hsa_circ_0004276 was negatively correlated with BMD in PMO patients and demonstrated high sensitivity and specificity for PMO diagnosis. This finding is based on an expanded cohort of 115 PMO patients and 108 age- and gender-matched healthy controls. Furthermore, hsa_circ_0004276 inhibited BMSC OD by interacting with the RBP ELAVL1.

In the bioinformatics analysis, the authors discovered that 10 of the top 20 circRNAs with the largest Log2 fold change were not cataloged in the circbank database. This highlights a challenge in bioinformatics, where database completeness and update frequency may lag behind scientific discoveries. These unannotated circRNAs may play significant roles in PMO, and future studies should prioritize their experimental validation to confirm their existence and elucidate their functions in PMO. Previous research has established the diagnostic potential of circRNAs in PMO, such as hsa_circ_0001445.^27^ The present study identifies hsa_circ_0004276 as a novel diagnostic marker for PMO, with ROC curves demonstrating its high sensitivity and specificity. However, the authors acknowledge that the diagnostic utility of hsa_circ_0004276, as indicated by the AUC, requires validation in larger, independent cohorts, particularly those including early-stage PMO patients and individuals at varying fracture risks. Additionally, hsa_circ_0004276 exhibited resistance to digestion by RNase R and actinomycin D, underscoring its structural stability, which is advantageous for maintaining consistent levels in biological samples. In PMO patients, hsa_circ_0004276 showed a strong negative correlation with BMD, suggesting its potential as a biomarker for monitoring disease progression and therapeutic efficacy. Nevertheless, it remains unclear whether the differential expression of hsa_circ_0004276 is more pronounced in early or late stages of PMO, necessitating further investigation with samples from patients at various disease stages.

Impaired OD capacity of BMSCs leads to reduced osteoblast numbers and function, thereby promoting PMO development.^28^ The authors observed that hsa_circ_0004276 expression decreased in a time-dependent manner during BMSC OD, suggesting its regulatory role in this process. However, it is unclear whether hsa_circ_0004276 continues to influence osteoblast viability and apoptosis after BMSCs have differentiated into osteoblasts, a question that warrants exploration in future studies. Notably, there is a significant research gap in understanding RBP-circRNA interactions in PMO. For instance, CircZNF367 has been shown to maintain CRY2 mRNA stability by interacting with FUS, thereby promoting osteoclast differentiation and osteoporosis.^29^ Similarly, CircPlod2 enhances OD of BMSCs by destabilizing Mpo mRNA through its interaction with IGF2BP2.^30^ In thisstudy, knockdown of hsa_circ_0004276 significantly promoted OD of BMSCs, a mechanism mediated by the downregulation of ELAVL1 protein expression. ELAVL1 is an RBP known to inhibit OD by targeting and regulating DMT1.^21^ RIP experiments confirmed the interaction between ELAVL1 and hsa_circ_0004276, and knockdown of hsa_circ_0004276 led to a marked reduction in ELAVL1 protein levels. The authors hypothesize that hsa_circ_0004276, upon binding to ELAVL1, alters the protein’s three-dimensional structure, potentially shielding it from proteolytic degradation. However, DMT1 is likely only one of several downstream targets of the hsa_circ_0004276/ELAVL1 axis, and given ELAVL1’s ability to interact with multiple mRNAs, extensive future work is needed to fully elucidate this regulatory network.

This study underscores the pivotal role of circRNAs in regulating bone metabolic diseases, particularly PMO. hsa_circ_0004276 emerges as a novel biomarker with high stability and diagnostic potential, offering a new avenue for PMO detection. Its negative correlation with BMD also positions it as a valuable tool for monitoring treatment efficacy and disease progression. Future research should investigate the expression patterns of hsa_circ_0004276 across different stages of PMO and evaluate its utility in assessing therapeutic responses. Moreover, considering the involvement of circRNAs in various diseases, integrating hsa_circ_0004276 with other established biomarkers could enhance the precision and sensitivity of PMO diagnosis. Further studies should also explore the role of hsa_circ_0004276 in other bone metabolic disorders to broaden its clinical applications. Additionally, the interaction between RBPs and circRNAs in PMO pathogenesis requires deeper investigation, and laboratories should focus on validating the biological functions and mechanisms of newly identified circRNAs, especially those not yet widely reported.

Despite these promising findings, the present study has limitations. First, while the authors have expanded the cohort size, the stability of circRNAs in serum and their biological origin ‒ whether they fully reflect the skeletal status ‒ remain uncertain. The relationship between serum levels of hsa_circ_0004276 and its expression in bone tissue or BMSCs also needs further clarification. Second, the diagnostic potential of hsa_circ_0004276 must be validated in diverse populations, including early-stage PMO patients and those with varying fracture risks, to ensure its clinical applicability. Third, longitudinal data are essential to understand the dynamic changes of hsa_circ_0004276 during PMO progression and in response to therapy.

To enhance the clinical relevance of hsa_circ_0004276, future studies should aim to correlate its levels with fracture incidence and treatment outcomes, such as responses to bisphosphonates. Additionally, comparing hsa_circ_0004276 with existing PMO biomarkers like CTX and P1NP will help define its unique clinical value, particularly whether it provides insights into the bone marrow microenvironment or BMSC function beyond traditional measures of bone turnover. Given the in vitro findings, exploring hsa_circ_0004276 as a therapeutic target is highly promising. For instance, testing antisense oligonucleotides targeting hsa_circ_0004276 in PMO animal models could validate its therapeutic potential. Furthermore, developing accessible detection methods, such as ELISA for circRNA quantification, would facilitate its translation into clinical practice.

In conclusion, this work highlights the importance of circRNAs in PMO regulation and identifies hsa_circ_0004276 as a potential diagnostic biomarker and therapeutic target.

## Availability of data and materials

The datasets used and/or analyzed during the present study are available from the corresponding author on reasonable request.

## Ethics approval

The present study was approved by the Ethics Committee of Chifeng Municipal Hospital (n° 202006CF3254) and written informed consent was provided by all patients prior to the study start. All procedures were performed in accordance with the ethical standards of the Institutional Review Board and The Declaration of Helsinki, and its later amendments or comparable ethical standards.

## Authors' contributions

BaiQuan Fu designed the research study. BaiQuan Fu and YuXin Sui performed the research. Yu Liu and XiaoTian Yang provided help and advice. Yu Liu, XiaoTian Yang and Hui Leng analyzed the data. BaiQuan Fu wrote the manuscript. Hui Leng reviewed and edited the manuscript. All authors contributed to editorial changes in the manuscript. All authors read and approved the final manuscript.

## Declaration of competing interest

The authors declare no conflicts of interest.

## Appendix Supplementary materials

Image, application 1
